# Predicting Stage-Specific Recurrent Aberrations From Somatic Copy Number Dataset

**DOI:** 10.3389/fgene.2020.00160

**Published:** 2020-02-26

**Authors:** Chaima Aouiche, Bolin Chen, Xuequn Shang

**Affiliations:** ^1^School of Computer Science, Northwestern Polytechnical University, Xi'an, China; ^2^Key Laboratory of Big Data Storage and Management, Northwestern Polytechnical University, Xi'an, China; ^3^Centre for Multidisciplinary Convergence Computing, School of Computer Science, Northwestern Polytechnical University, Xi'an, China

**Keywords:** cancer evolution, somatic copy number alteration, aberrant genes, pathological stages, pathway interaction network

## Abstract

Exploring the evolution process of cancers and its related complex molecular mechanisms at the genomic level through pathological staging angle is particularly important for providing novel therapeutic strategies most relevant to every cancer patient diagnosed at each stage. This is because the genomic level involving copy number variation (CNV) has been recognized as a critical genetic variation, which has a large influence on the progression of a variety of complex diseases. Great efforts have been devoted to the identification of recurrent aberrations, single genes and individual static pathways related to cancer progression. However, we still have little knowledge about the most important aberrant genes related to the pathology stages and their interconnected pathways from genomic profiles. In this study, we propose an identification framework that allows determining cancer-stages specific patterns dynamically. Firstly, a two-stage GAIA method is employed to identify stage-specific aberrant copy number variants segments. Secondly, stage-specific cancer genes fully located within the aberrant segments are then identified according to the reference annotation dataset. Thirdly, a pathway evolution network is constructed based on the impacted pathways functions and their overlapped genes. The involved significant functions and evolution paths uncovered by this network enabled investigation of the real progression of cancers, and thus facilitated the determination of appropriate clinical settings that will help to assess risk in cancer patients. Those findings at individual levels can be integrated to identify robust biomarkers in cancer progressions.

## 1. Introduction

Somatic copy number alterations (SCNAs) are one of the prevalent forms of genetic variations which play important roles in the progression of numerous diseases, such as cancers (Zack et al., 2013; Heitzer et al., 2016). SCNAs have much clinical relevance compared to other genetic alterations, and they can be good markers of cancer genome aggressiveness (Heitzer et al., 2016). Hence, the identification of specific signatures from CNAs will shed light on elucidating the complex mechanisms behind cancers evolution, and therefore lead to a promotive development in cancer treatment strategies (Lowe et al., 1994; Tsao et al., 2005; Kim et al., 2008; Cheang et al., 2009).

The evolution of cancers involves many complex and dynamic cellular processes that can be precisely described through pathological stages, which are often divided into several stages, from the initial stage to the later deleterious stage. Where cancers at early appearance (stage I or II) are typically viewed as treatable; however, many more aggressive and active therapies would be needed as they developed to harmful stages (stage III or IV). Thus, there was a critical need toward the extraction of reliable biomarkers characterizing the dynamics associated with these stages, including (1) stage-specific recurrent SCNAs, (2) their related aberrant genes, and (3) their enriched dysfunctional pathways (Chen et al., 2009, 2010; Lee et al., 2016; Liang et al., 2016; Wang et al., 2016; Nibourel et al., 2017; Zhu et al., 2017).

Recent developments on high-throughput genomic technologies have generated diverse tumor datasets with various clinical/pathological stages, conditions or tissues, for which CNAs and other omics-data have been collected. They provide effective ways to identify different biological patterns including individual genes, pathways, specific loci and individual chromosomal regions. However, the majority of these proposed ways completely ignore the topology and the interaction between these patterns, as well as their specificity along with the pathology stages. Since specific genes and pathways extracted from these stages across different regions will often act together in complex systems (Karczewski and Snyder, 2018; Ma et al., 2018), whose dynamic events are the results of multiple complex interactions that help to extract useful dynamic cellular functions, and that can well illustrate the progression and metastasis of cancers.

Fortunately, the usage of biological networks/pathways has turned out to be an effective method to describe the details of the dynamic changes and functional mechanisms associated with the individual stages of cancers, where individual nodes represent biological entities, i.e., genes or pathways, and each edge corresponds to an interaction between a pair of nodes. Those biological networks include but not limited to cellular pathways, gene regulation networks (Vaquerizas et al., 2009), protein-protein interaction networks (Schwikowski et al., 2000), and many disease related networks (Menche et al., 2015). Such networks can be efficiently used to investigate the dynamic biological activity behind cancers evolution.

A suite of well-established algorithms has also been proposed at the chromosome level to accurately detect recurrent SCNAs (Morganella et al., 2011), to investigate multiple cancer stages (Xia et al., 2004), or to use gene expressions to analyze the evolution processes of cancers.

To further extend the study to individual cancer stages, we propose an analysis framework to elucidate the dynamic evolution processes of cancers. Firstly, the recurrent aberrations associated with cancer-specific stages were discerned through (a) the identification of occurring sequential changes moving from stage I to stage IV and (b) the determination of correlations between higher frequency of CNA and the higher aggressive stage. Secondly, the stage-specific cancer related genes were carefully detected via the obtained CNV information. Thirdly, the stage-specific pathways were extracted and a pathway interaction network was generated by connecting functional pathways in adjacent stages. The remainder of the paper includes three sections: section 2 discusses the data sources and the methodology used in the identification framework, section 3 reports the results, and section 4 provides the conclusion of the study.

## 2. Materials and Methods

### 2.1. Data Collection and Grouping

Clinical and Somatic copy number alteration (by SNP 6.0 array) datasets on Level3 colorectal cancer (COADREAD) were downloaded from the Broad GDAC Firehose^1^.

Somatic copy number alteration (SCNA) minus germline SCNA was produced using GISTIC 2.0 and then divided into four groups based on the available clinical information of the same group of clinical patients. From clinical data, we take only the patients with available “pathology *t* stage” information, which defines the diagnosis stage of individual samples (*t*_1_, *t*_2_, *t*_3_, and *t*_4_). For the sample collection, we count the number of patients in the four *t* stages. Those individual samples with pathological information were aligned to the corresponding SCNA samples to get their copy number information for our following analysis.

Finally, 219 samples (*t*_1_ = 9, *t*_2_ = 46, *t*_3_ = 145, and *t*_4_ = 19) retained from clinical data were mapped to 47,140 samples from SCNA data (*t*_1_ = 1,255, *t*_2_ = 9,232, *t*_3_ = 32,293, and *t*_4_ = 4,360), respectively, and used to conduct our subsequent analysis. These details are shown in Table 1.

**Table 1:** The clinical and CNV datasets information from Broad Firehose TCGA project.

**Pathology stages**	**Clinical samples**	**CNV samples**
Pathology_*t*_1_	9	1,255
Pathology_*t*_2_	46	9,232
Pathology_*t*_3_	145	32,293
Pathology_*t*_4_	19	4,360

In addition, for recurrent CNAs identification from pre-computed GISTIC 2.0 SCNA data, GAIA (Morganella et al., 2011) with FDR *Q* < 0.10 was applied separately for each pathology stage using ten iterations. For genomic SCNA gains and losses plotting, an R script was used with a cut-off also specified at FDR *Q* < 0.10. For the genes annotation of the recurrent SCNA regions, the biomaRt (Durinck et al., 2005) and GenomicRanges (Lawrence et al., 2013) packages available through Bioconductor of R Studio were considered.

For the network construction, pathways were extracted from the Reactome database^2^. Since pathways with a smaller number of genes may lack significant biological knowledge, we collected, in this study, a set of pathways by filtering those with five genes. We ended up with 447 impacted pathways.

### 2.2. Stage-Specific Related Recurrent Somatic Copy Number Alteration Regions Identification

To identify the recurrent SCNA for the series of the pathological stages separately, a two-stage GAIA (genomic analysis of important aberrations) method (Morganella et al., 2011) was performed to determine the most significant recurrent CNA for the four pathology stages. In particular, this method follows two main steps: Significance testing and Homogeneous peel-off, to identify the most significant independent regions where a discrete representation of data is mainly considered.

Based on that, we first build a CNV matrix of regions using probes meta file from GISTIC 2.0 (available at^3^). Then, we define the recurrent CNA by FDR *Q* < 0.10 using ten iterations. Finally, we generate the genomic plots of the four stages using a GAIA plot function in R Studio, with the cut-off set also to FDR *Q* < 0.10.

**Figure 1:**
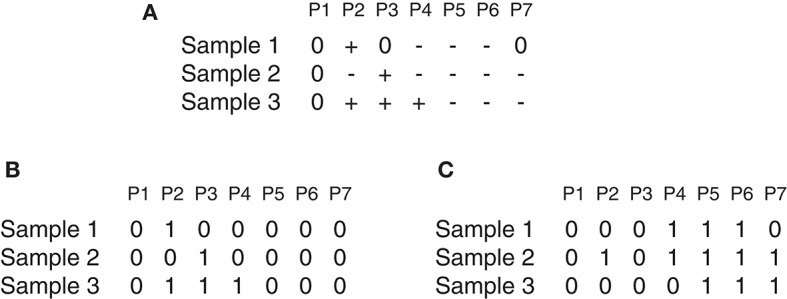
GAIA illustrative example. **(A)** Represent an example of matrix A, where + denotes gain, − denotes loss and 0 denotes no alteration. **(A)** Contain two homogeneous regions from probes P4 to P6 for samples S1 and S2 and from probes P5 to P7 for samples S2 and S3. **(B,C)** Show the matrices AL and AD determined of the matrix in **(A)**.

**Figure 2:**
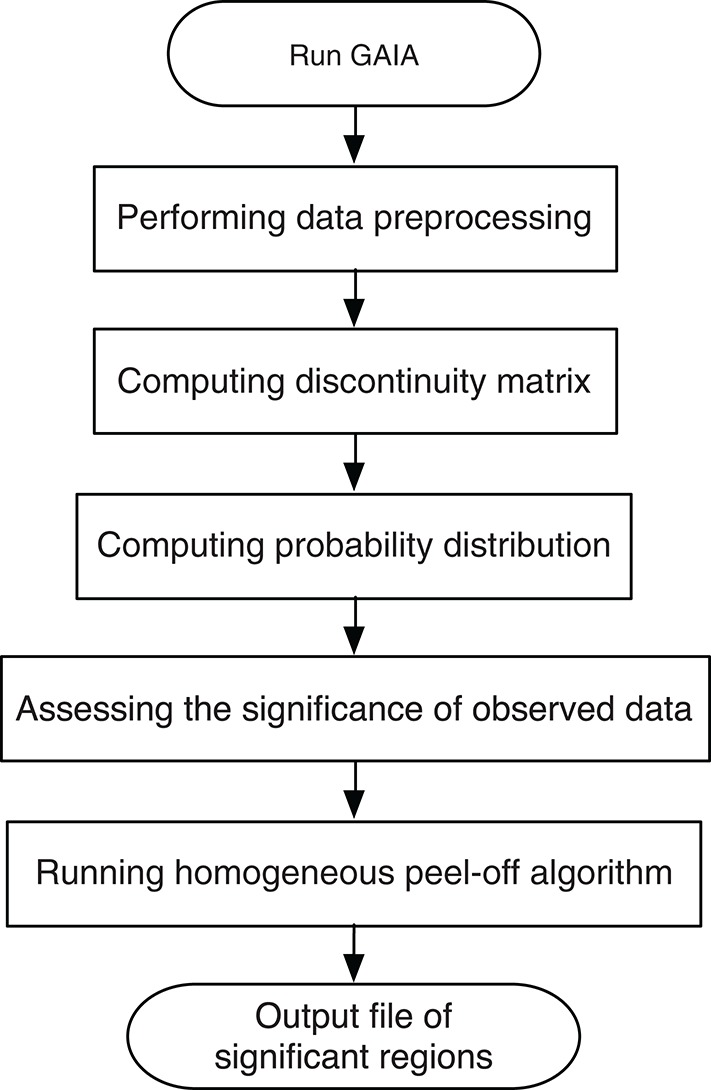
The flow chart of the GAIA method implementation steps.

Suppose there is a set of *N* samples (patients) and *M* observed probes, the data can be arranged as an *N* × *M* dimension matrix A. As an illustrative example (Figure 1), A can represent a chromosome of seven observed probes and three samples. The matrix *A* can be split into two matrices *AL* and *AD* where each element *a*_*ij* ∈ *AL*(*AD*) *i* = 1, …, *N* and *j* = 1, …, *M* can be denoted either by 1 as a gain (or loss) found in the *j*_*th* marker of the *i*_*th* sample, or by 0 otherwise as shown in Figures 1B,C, which represents the matrices *AL* and *AD* determined from the matrix *A* reported in Figure 1A. Three major steps can be applied to this matrix (gain or loss interest) to identify the significant peaks and omit the spurious peaks in a region based on *q*-values configuration, *h*-values calculation and multiple iterations. More details are described here and depicted in Figure 2.

First, a permutation test is performed on every individual marker to compute the probability distribution, so that we can estimate the statistical significance of the observed data.

Second, in order to define the homogeneous regions, we focus on the state of every paired adjacent markers (*j* and *j* + 1) rather than a single marker, and we calculate the degree of homogeneity between them. Given a matrix *H* of size (*N* × *M* − 1), with an element *H*_*ij*_ that has the value of 0 for maximum homogeneity, or the value of 0.5 for a medium homogeneity, or the value of 1 for a minimum homogeneity. From this matrix, we can obtain overall information on the homogeneity of the dataset based on the (*h*-value) that can be computed as follow:

(1) $$\begin{array}{l} h_{j}=\frac{1}{N}\overset{N}{\underset{i=1}{\sum }}H_{ij},\;j=1,\ldots ,M-1 \end{array}$$

Third, an iterative peel-off procedure is carried out on the matrix *H* by expanding the left and right boundaries of the region until the following conditions are satisfied. The left boundary expanded if:

(2) $$\begin{array}{l} q_{l-1}\leq q_{thr}\;\text{AND}\;h_{l-1}\leq h_{thr} \end{array}$$

and the right boundary expanded if:

(3) $$\begin{array}{l} q_{r+1}\leq q_{thr}\;\text{AND}\;h_{r}\leq h_{thr} \end{array}$$

where *l* and *r* denote the left and the right boundary of the peak with minimum *q*-value, with 1 ≤ *l*, *r* ≤ *M*, while *h*_*thr* represents a significance threshold value for homogeneity measurement. This value can take 0, 1, or values between 0 and 1.

**Figure 3:**
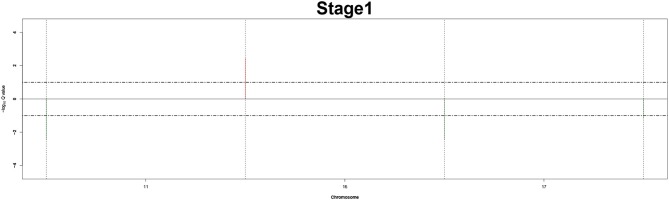
Recurrent genome-wide SCNAs in stage 1. Genome-wide amplifications (red blocks) and deletions (green blocks) in stage 1.

**Figure 4:**
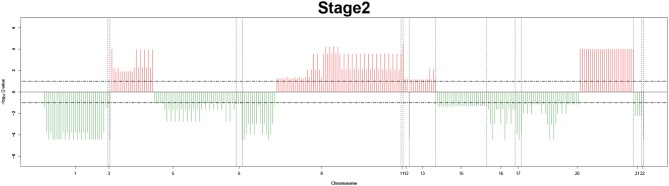
Recurrent genome-wide SCNAs in stage 2. Genome-wide amplifications (red blocks) and deletions (green blocks) in stage 2.

**Figure 5:**
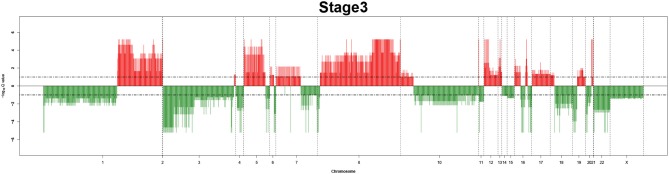
Recurrent genome-wide SCNAs in stage 3. Genome-wide amplifications (red blocks) and deletions (green blocks) in stage 3.

**Figure 6:**
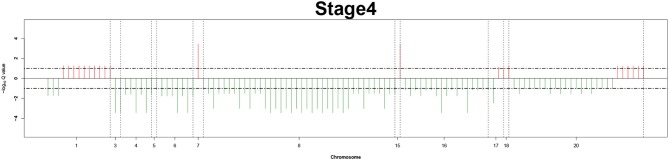
Recurrent genome-wide SCNAs in stage 4. Genome-wide amplifications (red blocks) and deletions (green blocks) in stage 4.

Remarkably, large recurrent SCNAs have been produced in this study at different chromosome positions moving from pathology_*t*_1_ to pathology_*t*_4_. More details are shown in Figures 3–6, respectively, which summarize the frequencies of the four pathology stages.

### 2.3. Stage-Specific Related Aberrant Genes Identification

The second essential step allowing a comprehensive elucidation of the cancer evolution process after SCNA regions identification is to identify the corresponding signature genes for individual stages. Therefore, the aberrant recurrent regions obtained previously at every pathology stage were then annotated to retrieve the genes that were significantly amplified or deleted. Using the reference annotation dataset of genes of biomaRt (Durinck et al., 2005), the final set of genes at *cut-off* = 0.10 with the precise co-ordinates regions from human genes in which it was found to have CNA, have been obtained. Further details are shown in Table 2, which lists the total number of genes selected in the four pathology stages.

**Table 2:** The number of aberrant genes and enriched pathways detected at each pathology stage.

**Pathology stages**	**Defined # of genes**	**# Of aligned pathways**
Pathology_*t*_1_	423	5
Pathology_*t*_2_	3,265	110
Pathology_*t*_3_	8,500	447
Pathology_*t*_4_	2,244	94

### 2.4. Stage-Related Pathways Extraction

After obtaining the deviant amplified or deleted genes at every pathology stage, the given genes were aligned to pathways on the basis of the biological pathways in the Reactome database from which a total of 3,305 was collected. The pathways found include clusters of pathways from different pathologies: 396 pathways from pathology_*t*_1_, 895 pathways from pathology_*t*_2_, 1,218 pathways from pathology_*t*_3_, and 796 pathways from pathology_*t*_4_.

As long as a single gene can be assigned to different pathways, and the latter would consist of a different number of genes, we set the study sample to every pathology's pathways consisting of genes whose size is >5. This is due to the fact that pathways with fewer genes would have limited biological content (Ahn et al., 2014). Therefore, a total of 656 pathways (*t*_1_ = 5, *t*_2_ = 110, *t*_3_ = 447, *t*_4_ = 94) was collected (Table 2). Finally, duplicated pathways were omitted, and only pathways that occurred in at least two pathological stages were extracted and considered as our stage-specific pathways to be further analyzed.

### 2.5. Pathway Evolution Network Construction

After identifying the signature genes for each stage and after extracting and integrating their specific Reactome pathways, they are pooled together, their terms are unified, and their official annotated pathway descriptions are obtained from the database. Next, a pathway interaction network related to SCNA is constructed where each node represents a biological specific pathway, and if the two pathways share common genes, then they are connected.

To clearly illustrate the dynamic evolution process through this pathway network, specific colors were used to evince the pathways that get evolved between the four individual stages, and the width of edges is applied to indicate the strength of associations between them. The width was calculated using an overlap score defined as:

(4) $$\begin{array}{l} W=\frac{k^{2}}{p*q}. \end{array}$$

where *k* represents the number of the overlapping genes between a pair of pathway *P*_*i*_ and pathway *P*_*j*_, *p* and *q* stand for the total numbers of genes in *P*_*i*_ and *P*_*j*_, respectively.

## 3. Results and Discussions

### 3.1. Stage-Related Recurrent Genome-Wide SCNAs Frequencies

The recurrent CNAs from four pathology stages were identified by investigating the sequential changes from pathology_*t*_1_ to pathology_*t*_4_ according to their different frequencies. This is based on the assumption that higher frequency of CNA will correlate with higher cancer stages. In fact, large genomic differences in recurrent SCNAs were observed in each pathology stage. Figures 3–6 represent the genome-wide amplifications and deletions of the four pathology stages, which generated with cut-off defined at FDR *Q* < 0.10. To be more specific, there were no significant segments in stage 1, but for stage 2, stage 3, and stage 4, the most of their regions were significantly amplified or deleted.

Moreover, more aberrant chromosomes get involved in these three stages. The frequency of aberrant segments were higher in stage 2 than in stage 1, and it kept increasing in stage 3. For example, stage 1 involved only three abnormal chromosomes with very low frequency. However, stage 2 and stage 3 involved more abnormal chromosomes segments with higher frequencies of amplifications or deletions. A clear evolution process of cancer could be observed by connecting those major chromosomal abnormalities stage-by-stage.

### 3.2. The Number of Stage-Specific Related Genes

The amplified and deleted genes which fully located within the aberrant regions of the four pathological stages were detected by using the biomaRt and the GenomicRanges packages in R (Table 2), wherein a total of 423, 3,265, 8,500, and 2,244 genes were identified as representative signature genes in stage 1, 2, 3, and 4, respectively. All of these potential candidate genes were carried out for pathway network generation and functions interpretation, due to their ability to effectively explore cancer progression.

### 3.3. Dynamic Pathway Interaction Network Generation and Visualization

**Figure 7:**
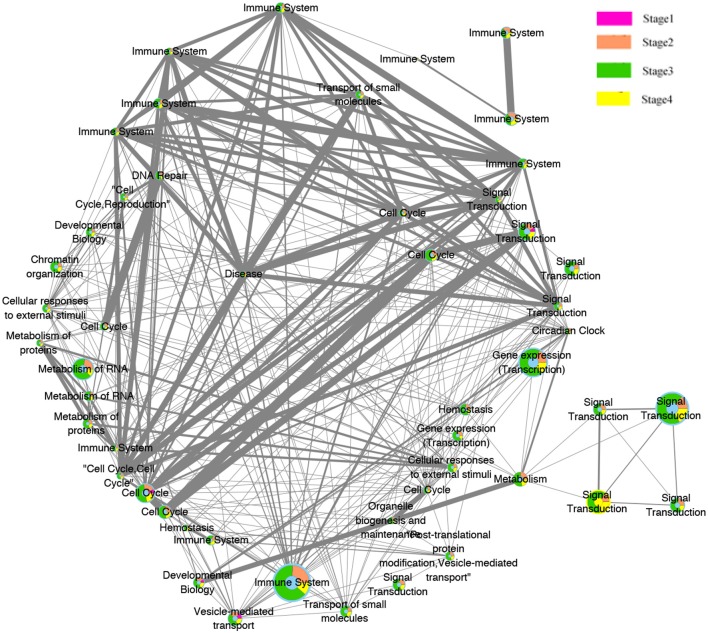
Pathway functions interaction network. Node illustrate the biological pathways function. Edges illustrate the relationships between the functions at the adjacent stages. The size of the node is proportional to the number of genes in the pathway, The thicker the edges, the more overlapped genes between pathways of the adjacent stages. The color of pink, orange, green, and yellow inside the nodes indicate the pathways functions belongs to the four stages.

The evolution network was generated by considering the enriched pathways as nodes, and the overlapping genes in two corresponding pathways as edges. The network contains 50 nodes and 339 edges. Different colors (pink, orange, green, yellow) were used to showcase how these pathways evolved across the four pathologies adjacent stages, whereas the width of edges indicated the strength of their connections. The network was then visualized by Cytoscape software, where the different significant evolution paths are shown. These further details are depicted in Figure 7.

### 3.4. Stage-Related SCNAs Pathways Specific Functions Interpretation

The substantial analysis in this study confirmed the efficacy of the proposed framework. The detected genes were first enriched in many important pathways and these pathways, in turn, were strongly related to many critical cellular functions, such as cell cycle, disease, gene expression (Transcription), immune system, neuronal system, signal transduction, and metabolism of proteins and RNA. Some extra extremely enriched pathways obtained from both the amplified genes and deleted genes are shown in Table 3.

**Table 3:** The pathway enrichment of both the amplified and deleted genes from each pathology stage.

**Pathway**
DNA repair
Transport of small molecules
Developmental biology
Programmed cell death
Cell-cell communication
Hemostasis
Post-translational protein modification
Cellular responses to external stimuli

Interestingly, most of these functions were related to the immune system. This preliminary investigation can be clearly seen from the evolution network depicted in Figure 7. In this network, most of the pathways-related immune system were strongly related to each other with thicker edges. Furthermore, since the pathways enriched from the deleted genes (2,630 pathways) were higher than those of the amplified genes (2,069), the genes annotated in them were probably dynamically changed with the four pathological stages as can be observed from the evolution paths of the constructed evolution network. This dynamic change may lead to decrease the immunity in colorectal cancer and thus to homeostasis perturbance. Therefore, increasing the immunity activities across the stages will be effective and beneficial for many cancer types.

Moreover, signal transduction and cell cycle were also highlighted here. These functions are invariably perturbed in cancer since they are essential in regulating, activating multiple cellular process and signaling molecules. They can induce cell proliferation, differentiation, and survival of various cancers (Cao et al., 2014).

These functions were also involved in diverse human and animal diseases, and they provide useful information to understand the initiation and progression of many complex diseases.

## 4. Conclusion

Complex diseases evolution process is too difficult to be inferred by single genes, individual pathways or even a type of genomic data. However, understanding this evolution mechanism at a single level can be leveraged to identify more robust biomarkers and valid biological functions when integrating it with other genomic levels.

CNAs hold a very important role in cancers. Therefore, finding the recurrent CNA from cancer specific stages is a promising task for identifying their essential driver events. We have proposed to investigate the key indicators associated with cancer progressions by: (1) identifying the sequential changes/chromosomal abnormalities related to these stages, (2) defining their significant key genes, and (3) generating an evolution network rather than gene networks.

We have also used an interesting rCNA-algorithm that has the ability to identify many significant recurrent regions, due to its powerful homogeneous peel-off and its parameter setting that is very straightforward.

These critical factors identified from this valid alternative method enabled us to identify the differences between the molecular portraits of the different pathological stages, and improved our understanding of the pathogenesis and underlying molecular mechanism related to cancer initiation and progression. Moreover, the aberrant candidate genes and pathways characterized every pathology stage identified here could give us a clue to specific therapeutic targets for treatment of cancers.

In summary, such findings at a single level will help decide which types of omics data and methodologies will be better integrated to improve clinical research endpoints, and therefore get insights into the serious issues driving complex diseases. Furthermore, an interesting work would be to not only compare CNA events between cancer stages, but to also link these to somatic mutations in CIN (chromosomal instability) signature genes.

## Data Availability Statement

Publicly available datasets were analyzed in this study. This data can be found here: (1) FireBrowse: http://firebrowse.org, (2) Reactome database: http://www.reactome.org, (3) CNV data: ftp://ftp.broadinstitute.org/pub/GISTIC2.0/hg19support/.

## Author Contributions

BC initialized this study. CA and BC discussed many times to finalize the work plan. XS gave suggestions many times to modify this study. CA conducted the numerical experiments and drafted the manuscript. All authors read the manuscript and revised it, and agreed with the final version.

### Conflict of Interest

The authors declare that the research was conducted in the absence of any commercial or financial relationships that could be construed as a potential conflict of interest.

## Footnotes

^1^http://firebrowse.org/

^2^http://www.reactome.org

^3^ftp://ftp.broadinstitute.org/pub/GISTIC2.0/hg19support/
